# High-Performance Terahertz Photodetectors Based on Spiral Structure-Regulated Graphene

**DOI:** 10.3390/s26092633

**Published:** 2026-04-24

**Authors:** Lei Yang, Bohan Zhang, Yingdong Wei, Hongfei Wu, Zhiyuan Zhou, Zhaowen Bao, Huichuan Fan, Xiaoyun Wang, Lin Wang, Xiaoshuang Chen

**Affiliations:** 1 School of Microelectronics, Shanghai University, 20 Chengzhong Road, Shanghai 201899, China; yangl23@shu.edu.cn; 2 School of Information Science and Technology, ShanghaiTech University, Shanghai 201210, China; zhangbh2023@shanghaitech.edu.cn; 3 State Key Laboratory of Infrared Physics, Shanghai Institute of Technical Physics, Chinese Academy of Sciences, 500 Yu Tian Road, Shanghai 200083, China; weiyd@shanghaitech.edu.cn (Y.W.); fanhuichuan23@mails.ucas.ac.cn (H.F.); wangxiaoyun23@mails.ucas.ac.cn (X.W.); 4 School of Physical Science and Technology, ShanghaiTech University, Shanghai 201210, China; wuhf2023@shanghaitech.edu.cn (H.W.); baozhw2023@shanghaitech.edu.cn (Z.B.); 5 School of Physics, Donghua University, Shanghai 201620, China; zhouzhou66321057@163.com

**Keywords:** terahertz technology, photodetector, graphene, spiral antenna

## Abstract

**Highlights:**

**What are the main findings?**

- We designed a novel, room-temperature terahertz (THz) photodetector that leverages monolayer graphene coupled with a precisely designed counterclockwise spiral antenna.
- The device achieves exceptional sensitivity with a minimum noise equivalent power (NEP) of 80.7 pW/√Hz, a high zero-bias responsivity of 3.11 mA/W, and a rapid response time of less than 11 μs.

**What are the implications of the main findings?**

- By systematically analyzing the temporal response dynamics and using finite-difference time-domain (FDTD) simulations, we unambiguously identified the photothermoelectric (PTE) effect as the dominant operating mechanism.
- These findings provide a robust strategy and foundation for the development of high-performance, highly sensitive room-temperature THz optoelectronics.

**Abstract:**

Terahertz technology has demonstrated immense potential across a wide range of applications, particularly in the realm of THz photodetection. However, state-of-the-art detectors typically face fundamental trade-offs among sensitivity, response speed, operating temperature, and spectral bandwidth. While previous studies have shown that graphene field-effect transistors (GFETs) exhibit a broadband, room-temperature photoresponse to THz radiation—often attributed to photothermoelectric (PTE) and plasma-wave rectification effects—the similar functional dependence of these mechanisms on the gate voltage has historically made it challenging to disentangle their individual contributions. In this study, we leverage monolayer graphene as the photoactive material to overcome these limitations within a single device architecture. We present a novel THz photodetector driven predominantly by the PTE effect, facilitated by a precisely designed counterclockwise spiral antenna. The demonstrated device achieves exceptional room-temperature sensitivity, featuring a minimum noise equivalent power (NEP) of 80.7 $pW/\sqrt{Hz}$ alongside a rapid response time of less than 11 μs. Furthermore, by systematically analyzing the temporal response dynamics, we unambiguously identify the PTE effect as the dominant operating mechanism. These results provide a robust strategy for the development of high-performance, room-temperature THz optoelectronics, paving the way for advanced practical applications in high-capacity wireless communications and real-time THz imaging.

## 1. Introduction

The terahertz (THz) frequency band, spanning from 0.1 to 10 THz, has long been referred to as the “THz gap” [1]. The rapid advancement of modern technology has driven an increasing demand for the generation, detection, and manipulation of the THz spectrum. Bridging the gap between microwave electronics and infrared photonics [2,3], THz technology leverages unique optoelectronic characteristics that are highly sought after in fields such as wireless communications, spectroscopy, materials science, sensing, and imaging [4,5,6,7]. Over the past decade, active and passive optoelectronic devices have rapidly evolved, enabling disruptive applications in medicine, biology, security, astronomy, and real-time tomography [8,9,10,11,12,13,14]. Since the 1980s, the advent of ultrafast lasers and semiconductor technologies has significantly propelled the THz field. While the THz detector remains a critical system component, the inherently low energy of THz photons makes high-speed, highly sensitive detection exceptionally challenging. Ideally, THz detectors should exhibit high sensitivity, rapid response times, low power consumption, compatibility with readout integrated circuits (ROICs), and low manufacturing costs [15]. Currently, widely used devices include pyroelectric detectors [16], Golay cells [16], and Schottky diode detectors [17]; however, each suffers from inherent performance limitations. Consequently, photothermoelectric (PTE) THz detectors have emerged as highly promising room-temperature candidates capable of satisfying these stringent requirements.

PTE detectors operate by coupling photothermal and thermoelectric conversion processes. When the detector absorbs incident photons on one side, a localized temperature gradient is established. This gradient drives the spontaneous, directional diffusion of charge carriers from the high-temperature region to the low-temperature region, consequently generating an electric potential difference(ΔV). This phenomenon is known as the Seebeck effect [18], where the Seebeck coefficient (S) is defined as the ratio of ΔV to ΔT. The electromotive force generated by the PTE effect depends on the temperature gradient and the spatial variation in the Seebeck coefficient within the channel material:

(1) $$V_{PTE}=\int_{x_{L}}^{x_{R}}S\left(x\right)\nabla T\left(x\right)dx$$

where $x_{L}$ and $x_{R}$ represent the left and right sides of the channel, respectively. The Seebeck coefficient S, which depends on the Fermi energy ($E_{F}$), is described by the Mott relation [19]:

(2) $$S=-\frac{\pi^{2}k_{B}^{2}T}{3e}\frac{1}{\sigma }{\left.\frac{d\sigma }{dE}\right|}_{E=E_{F}}$$

where k_B_ is the Boltzmann constant, e is the elementary charge, σ is the electrical conductivity, and T is the absolute temperature. Lemme et al. first demonstrated a PTE detector based on a graphene p-n junction, utilizing sub-micrometer split gates to locally modulate the Seebeck coefficient; however, the peak responsivity remained below 1 mA/W [20]. In 2014, Cai et al. extended this detection scheme to the THz regime using an asymmetric electrode structure, which induced an asymmetric Seebeck profile via energy band bending. Despite this, optical phonon scattering during electron relaxation limited their responsivity to a relatively low 0.25 V/W, [21]. In 2019, Castilla et al. fabricated a gate-tunable graphene p-n junction PTE detector operating between 1.8 and 4.2 THz, achieving a peak responsivity of 105 V/W, a noise equivalent power (NEP) of 80 $pW/\sqrt{Hz}$, and a response time of less than 30 ns [15]. Subsequently, in 2020, Guo et al. reported a black phosphorus-based PTE detector with an ultra-short channel, employing dissimilar contact metals to induce spatial Seebeck asymmetry. This device achieved responsivities of 297 V/W and 135 V/W at 0.12 THz and 0.29 THz, respectively, alongside a response time of 2.3 ns and NEPs of 58 $pW/\sqrt{Hz}$ and 138 $pW/\sqrt{Hz}$ [22]. More recently, in 2021, Suzuki et al. utilized suspended carbon nanotubes to minimize thermal dissipation to the substrate. This enhanced the localized temperature gradient within the channel, yielding an NEP of 2.45 $nW/\sqrt{Hz}$ [23].

In this work, we propose a room-temperature terahertz (THz) photodetector based on the seamless integration of a counterclockwise spiral antenna with large-area monolayer graphene. Uniquely, this spiral structure can simultaneously serve as the source, drain, and gate electrodes. This monolithic design maximizes the spatial asymmetry of the localized electric field and generates a local temperature gradient under THz irradiation. In contrast to existing photothermoelectric (PTE) detectors that rely on structurally complex p-n junctions or heterogeneous metal contacts, our approach achieves highly efficient zero-bias photoresponse without the need for sophisticated external gate modulation. The proposed device architecture not only exhibits exceptional fundamental performance but also provides a reliable technological pathway for next-generation THz optoelectronics. This design strategy holds tremendous potential for a wide range of emerging practical applications, including high-speed wireless communications, non-destructive biomedical imaging, and advanced security sensing systems.

## 2. Methods

### 2.1. Device Fabrication

Large-area, monolayer graphene was synthesized via chemical vapor deposition (CVD) on copper foils. As established in pioneering studies [24,25], this growth process is widely recognized as a mature and highly reliable technique for producing high-performance optical and optoelectronic components. The self-limiting growth mechanism on copper ensures a continuous and uniform monolayer, which is essential for the reliability of THz sensing architectures. While the exact microscopic defect density was not quantitatively mapped for every individual device, Raman spectroscopy characterization of the transferred graphene consistently shows a negligible D-peak, with a representative intensity ratio of $I_{D}/I_{G}<0.1$. According to established quantitative models [26], such a low $I_{D}/I_{G}$ ratio corresponds to an exceptionally low microscopic defect density, ensuring high lattice integrity. This high structural quality is phenomenologically corroborated by the device’s exceptional macroscopic performance. Specifically, the microsecond-scale response time and robust zero-bias responsivity (as discussed in Section 3.3) indicate highly efficient, ultra-fast hot-carrier transport. A high defect density would induce severe carrier scattering and quench the photothermoelectric effect, rendering such performance physically unattainable. The synthesized graphene was subsequently transferred onto a high-resistivity Si/SiO_2_ substrate (high-resistivity silicon capped with 300 nm SiO_2_). The spiral antenna structures (Cr/Au, 20 nm/80 nm) were patterned directly onto the graphene using ultraviolet (UV) lithography followed by metallization. The active graphene channel was then defined through an additional photolithography step and oxygen plasma etching. Subsequently, a 30 nm thick Al_2_O_3_ dielectric layer was deposited via atomic layer deposition (ALD) to serve as the top-gate oxide. Finally, the top-gate electrodes were patterned using UV lithography.

### 2.2. Photocurrent Measurement

The electrical data was collected by a Keithley-4200 semiconductor parameter analyzer. The device response current to the THz wave was tested. To evaluate the broadband capabilities of the device, tunable THz sources covering three distinct frequency bands (0.02–0.04 THz, 0.08–0.12 THz, and 0.24–0.30 THz) were utilized. By scanning the incident frequencies within these bands, the peak photoresponse points were identified at approximately 0.04 THz, 0.12 THz, and 0.30 THz, respectively. Consequently, these three discrete peak frequencies were selected as representative testing points for detailed electro-optical characterizations. The photoresponse is recorded by a lock-in amplifier (LIA) and an oscilloscope after a low-noise voltage preamplifier. The detector responsivity (RA) is extracted from IPH through the relation $R_{A}=I/P\cdot S_{a}$, where $P=P_{in}/S$, $P_{in}$ is the incident power, and the area of the device to be tested is $S_{a}$, where $S_{a}=\lambda^{2}/4\pi$, S is the light spot. NEP is the figure of merit used to evaluate the performance of the device, and it can be estimated from $NEP=\nu_{n}/R$, where $\nu_{n}$ is the root mean square of noise voltage and *R* is voltage responsivity. To provide the lower limit of noise figure, both the thermal noise $\nu_{t}$ and shot noise $\nu_{b}$ were considered via $\nu_{n}=\sqrt{\nu_{t}^{2}+\nu_{b}^{2}}=\sqrt{4K_{B}T/r+2qI_{d}}$, where $K_{B}$ is Boltzmann constant, *T* is temperature, *q* is the elementary charge and $I_{d}$ is bias current or dark current of the device.

## 3. Results and Discussion

### 3.1. Device Structure and Characterization

**Figure 1:**
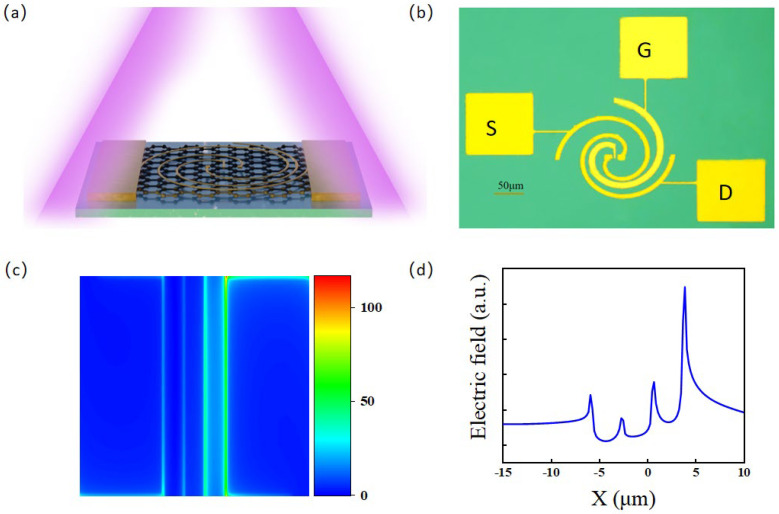
(**a**) Schematic illustration of the THz detectors and their electrical connection for the photoresponse measurement. (**b**) Structure of the spiral antenna optically coupled PTE device. (**c**) Simulated near-field distribution at 0.3 THz in the center of device. (**d**) The electric field about the center of the device.

The detector is based on monolayer graphene grown by chemical vapor deposition (CVD). The graphene was transferred onto a high-resistance Si/SiO_2_ substrate for subsequent antenna patterning (see Methods). A highly resistive Si substrate, rather than a heavily doped one, was explicitly chosen to eliminate the reflection loss of incident waves, as the THz wavelength is at least two orders of magnitude larger than the thickness of the dielectric layer. By integrating the spiral antenna directly as the contact electrodes, the spatial asymmetry within the channel is maximized, which is highly conducive to the formation of a localized temperature gradient (see Figure 1a). Due to the asymmetric electric field focusing induced by the antenna, one side of the graphene channel (typically at the electrode contact) absorbs a substantial amount of energy. Driven by ultrafast electron-electron scattering and exceptionally weak electron-phonon [27] (lattice) coupling in graphene, the localized electron gas is instantaneously heated to an elevated temperature, resulting in the generation of ‘hot electrons’. Consequently, the response is dominated by the photothermoelectric effect, and previously reported plasma-wave effects [28,29] are not considered in this work.

The left and right contacts serve as the source and drain, respectively, while the central contact functions as the gate. In Figure S1, these three terminals are directly coupled to the arms of the counterclockwise spiral antenna. For the inner spiral, the inner and outer radii are 9.5 μm and 99.5 μm, respectively. For the outer spiral, the corresponding radii are 17.5 μm and 107.5 μm. The top gate is electrically isolated from the graphene channel by an ultrathin (30 nm) Al_2_O_3_ dielectric layer. Following the antenna patterning and metallization/lift-off processes, oxygen-based plasma etching was performed to define the active graphene channel. Subsequently, electron-beam lithography (EBL) was utilized to pattern slits with varying spacing in the center of the channel. The respective terminals are labeled as S, D, and G in Figure 1b. A top-gated architecture was selected over a conventional back-gated design for two primary reasons: first, to avoid the severe electromagnetic wave attenuation caused by a highly doped back-gate; and second, to allow the antenna to effectively manipulate the near-field amplitude and distribution. When the graphene channel is positioned at the nanoscale focal point of the near-field, a non-uniform hot-carrier distribution is generated. This localized heating induces a potential gradient, facilitating the extraction of additional carriers upon the application of a source-drain bias.

**Figure 2:**
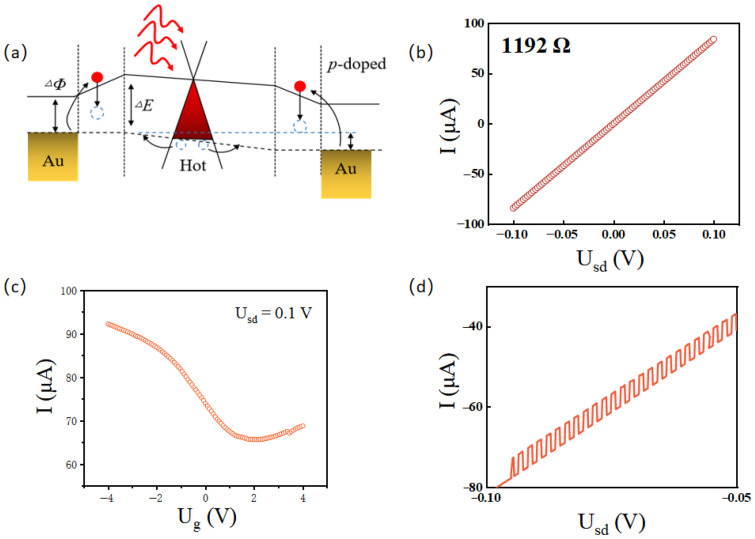
(**a**) Inherent physical mechanisms for device operation. (**b**) Room-temperature I-V curve. (**c**) Transfer characteristic curve under gate voltage. (**d**) I-V curve of the device under 1 Hz wave-free switching modulated illumination.

In high-performance hot-electron photodetectors, incident photons must possess sufficient energy to excite electrons over the potential barrier [30,31]. Notably, the inevitable energy losses typically associated with charge transfer in conventional hot-electron detectors can be effectively mitigated here due to the ultralow contact resistance at the graphene interface [32]. Consequently, the intrinsic hot-carrier dynamics within the graphene channel play a pivotal role in the detection mechanism, as illustrated in Figure 2a. Following initial photoexcitation, rapid electron-electron scattering causes the electron gas to undergo ultrafast heating. Because of the exceptionally weak electron-phonon coupling in graphene, the electron temperature can become significantly higher than the lattice temperature [33,34]. Although the initial momentum of these hot electrons is randomized, non-equilibrium diffusion drives a net excess electron flow from the heated contact region into the cooler graphene channel. Furthermore, this hot-electron transport can be directionally modulated by applying a small external bias (either via the top-gate or source-drain voltage, as depicted in Figure 2a).

### 3.2. Numerical Simulation of the Photothermoelectric Mechanism

The localized electric field distributions were simulated using the 3D Finite-Difference Time-Domain (FDTD) method. A normally incident THz plane wave was used as the excitation source, with Perfectly Matched Layers (PMLs) applied as boundary conditions. To accurately resolve the localized field asymmetry, a heavily refined local mesh was implemented at the antenna gaps. At the investigated THz frequencies, the metallic antennas were modeled as Perfect Electric Conductors (PECs) [35]. This approximation is highly accurate in the THz regime, as the extremely high conductivity and minimal skin depth of noble metals render field penetration and Ohmic losses negligible, yielding near-field profiles identical to those calculated using full dispersive Drude models [36] while significantly optimizing computational stability. Simulations provide a theoretical foundation by visually mapping the optical and electrical properties of the device.

The simulation model strictly replicated the actual geometric parameters of the fabricated device, including dimensions, vertical stacking, and antenna configuration. Additionally, perfectly matched layers (PMLs) were applied as absorbing boundary conditions to effectively suppress numerical reflections. This rigorous setup ensures high fidelity between the simulation environment and the experimental conditions.

Figure 1c illustrates the simulated electromagnetic field distribution near the device center under 0.3 THz irradiation. The incident THz wave exhibits strong spatial localization, which is fundamental to driving the photothermoelectric (PTE) effect. Specifically, the symmetry-breaking required for the PTE effect is induced via two primary mechanisms: (i) the inherent structural asymmetry of the metal antenna, and (ii) localized illumination. Both mechanisms disrupt the spatial equilibrium of the physical fields, establishing a non-uniform local electric field distribution. In addition, as shown in Figure S2, we also conducted a comparison between the forward and inverse helical antennas. Figure S3 presents the FDTD simulation comparisons of the gate positions of the helical antennas. The forward helical antenna with the gate located at the center is adopted in this paper.

Figure 1d maps the detailed local electric field profile within the device. A pronounced field enhancement and differentiation are observed near the asymmetric regions of the metal antenna. This highly localized field concentration generates a robust temperature gradient via the photothermal effect, which subsequently drives the asymmetric charge carrier distribution. Crucially, these FDTD simulation results are highly consistent with the experimental characterizations presented in Figure 2c, mutually corroborating the proposed PTE mechanism in our devices.

### 3.3. Optoelectronic Characterization and Device Performance

**Figure 3:**
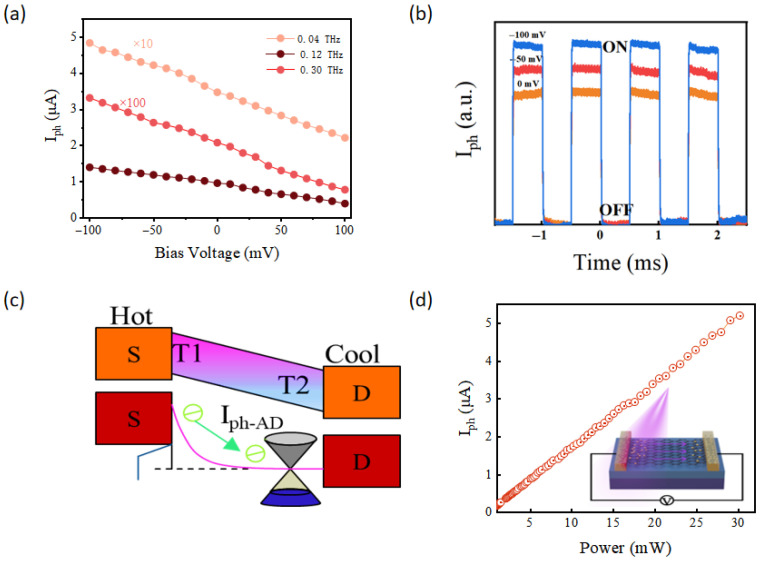
(**a**) Current voltage (I–V) characteristics of photodetectors based on graphene under different input frequency. (**b**) Characterization of the different power photoelectric performance of the device under 0.04 THz irradiation. (**c**) Photocurrent response due to hot-carrier diffusion from the hot to the cool side. (**d**) Power dependent optical response.

Figure 2b presents the I–V curves at various incident frequencies. The curves exhibit a highly linear trend, indicating that the metal-graphene contact interfaces do not introduce significant parasitic impedance. Furthermore, the transfer characteristic curve demonstrates that the device transitions from electron-dominated to hole-dominated conduction as it crosses the charge neutrality point (CNP). Figure 2d compares the I–V characteristics under dark and illuminated conditions. The illuminated curve reveals a photovoltaic-like response, which can be attributed to either the plasma wave rectification effect or the photothermoelectric (PTE) effect [37]. Due to the diffusion of charge carriers (holes) from the hot side to the cold side, as shown in Figure 3c, the net current flows along the channel without an electrically biased *U_sd_*. According to $∆T_{h}=∆U_{ph-SD}/S=I_{ph-SD}R_{SD}/S$ relationship, we can estimate the temperature rise after local photon absorption based on the zero bias photocurrent $I_{ph-SD}$, where $∆U_{ph-SD}$ is the open circuit photovoltage.

Figure 3a displays the bias-dependent photocurrent at 0.04 THz, 0.12 THz, and 0.3 THz. Due to the relatively weak responses at 0.12 THz and 0.3 THz, these data sets were scaled by a multiplication factor for clearer comparison. The maximum photocurrent is distinctly observed at 0.04 THz. Consequently, we evaluated the THz response waveforms at various incident power densities using a modulation frequency of 0.04 THz. As shown in Figure 3b, the THz response scales positively with incident power while the waveform shape remains well-preserved, consistent with our previous characterizations. Furthermore, Figure 3d confirms that the photocurrent exhibits a rigorous linear dependence on the incident power at 0.04 THz.

**Figure 4:**
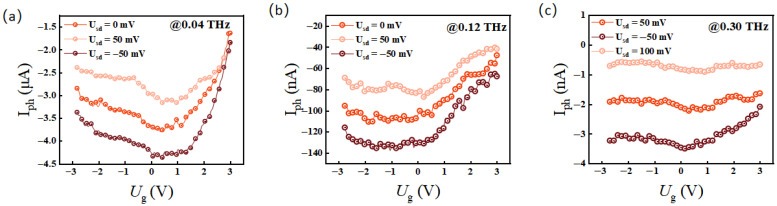
(**a**–**c**) Gate voltage *U_g_* dependence of photocurrent I_ph_ in devices with different bias voltage at 0.04 THz, 0.12 THz and 0.3 THz, respectively.

In Figure 4a, we extracted the photocurrent as a function of gate voltage (*U_g_*) at 0.04 THz under *U_sd_* biases of 0 mV, 50 mV, and −50 mV. These results align seamlessly with the observations in Figure 2c. Additionally, Figure 4b,c present the transfer characteristic curves at 0.12 THz and 0.3 THz, respectively, which are highly consistent with theoretical expectations.

Quantitatively, the gate-voltage-dependent photocurrent profiles in Figure 4 can be well-described by the analytical Mott relation. In a PTE-driven device, the photocurrent is given by $I_{ph}\left(U_{g}\right)=\left[S_{channel}\left(U_{g}\right)-S_{contact}\right]\Delta T/R\left(U_{g}\right)$. Based on the Mott formula, the Seebeck coefficient $S\left(U_{g}\right)$ is proportional to the logarithmic derivative of the conductance $\left(1/G\right)\left(dG/dU_{g}\right)$. The experimental $I_{ph}-U_{g}$ curves extracted in Figure 4 seamlessly trace the analytical derivative of the transfer characteristic curve shown in Figure 2c. The photocurrent exhibits pronounced extrema corresponding to the steepest slopes of the transfer curve and approaches a turning point near the charge neutrality point (CNP), providing robust quantitative validation that the PTE effect dominates the THz response.

Furthermore, it is highly instructive to compare our gating architecture with other existing advanced designs, such as the recently proposed terahertz photodetectors utilizing 2D ferroelectric materials (e.g., CuInP_2_S_6_) as the dielectric layer [38]. While ferroelectric gating offers substantial carrier modulation via out-of-plane polarization, it inherently introduces large hysteresis loops, which may complicate high-speed continuous detection due to memory effects. In contrast, our monolithically integrated top-gated spiral antenna yields a purely linear and hysteresis-free electrostatic modulation. More importantly, by leveraging the colossal local field enhancement at the asymmetric antenna gap, our device achieves a remarkably low NEP of 80.7 $pW/\sqrt{Hz}$, which is nearly an order of magnitude superior to the 0.64 $nW/\sqrt{Hz}$ (640 $pW/\sqrt{Hz}$) reported for state-of-the-art BP/CIPS ferroelectric detectors.

**Figure 5:**
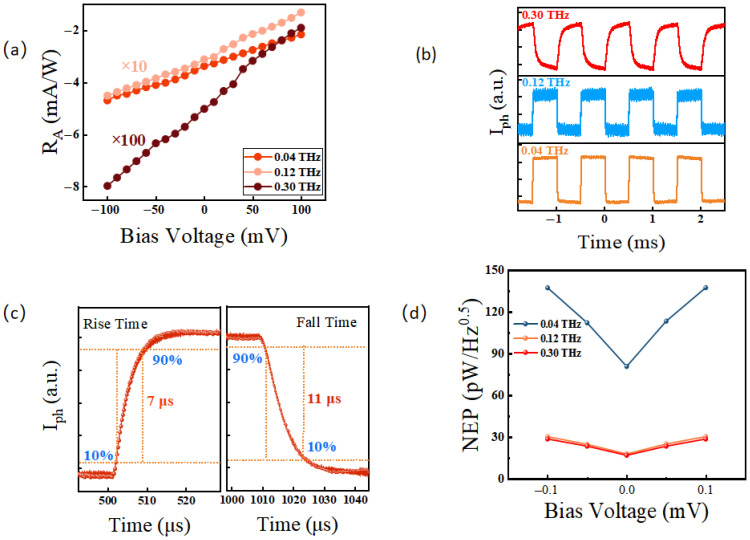
(**a**) The responsivity of the device. (**b**) Time-resolved light response waveform. (**c**) The rise time and fall time of the graphene photodetector at 0.04 THz. (**d**) Room-temperature NEP at different bias voltage.

Figure 5a shows the bias-dependent responsivity of the device. Notably, even at zero bias, the device achieves a high responsivity of 3.11 mA/W. To evaluate the response speed, the device was illuminated with fast on/off modulated THz radiation. Figure 5b displays the time-resolved photocurrent pulses at 0.04 THz, 0.12 THz, and 0.3 THz at zero bias. The pulse shapes are excellently preserved across all frequencies, demonstrating the device’s robust multiband detection capability. This broadband response perfectly matches the theoretical predictions for ultra-short channel architectures. As shown in Figure 5c, the response times—defined as the rise and fall times between 10% and 90% of the maximum signal amplitude—are approximately 7 μs and 11 μs, respectively. This microsecond-scale speed confirms that the response mechanism is predominantly photothermoelectric, verifying our initial hypothesis. Finally, Figure 5d illustrates the noise equivalent power (NEP) at different bias voltages. Accounting for the scaling factors used for the 0.12 THz and 0.3 THz data, the device achieves an outstanding minimum NEP of 80.7 $pW/\sqrt{Hz}$ at 0.04 THz. The comprehensive performance comparison of our device against other state-of-the-art active material detectors is summarized in Table 1.

As summarized in Table 1, the proposed spiral-antenna-coupled graphene photodetector exhibits highly competitive performance metrics, highlighted by a prominent zero-bias responsivity of 3.11 mA/W and a microsecond-scale response time (11 μs). The physical mechanisms underpinning its superiority over other state-of-the-art active-material detectors can be elucidated from both material and structural standpoints.

Materially, in contrast to 3D microporous graphene or macroscopic carbon nanotube networks—where prevalent grain boundaries and interfaces aggravate electron-phonon scattering and parasitic thermal dissipation—the large-area monolayer graphene utilized herein inherently features exceptionally weak electron-phonon coupling. This intrinsic characteristic enables the photoexcited electron gas to rapidly decouple from the crystal lattice, thereby establishing a pronounced non-equilibrium hot-carrier distribution that facilitates an ultrafast photoresponse. Furthermore, compared to black phosphorus, the zero-bandgap nature and linear Dirac dispersion of graphene facilitate highly efficient and accelerated hot-carrier dynamics, culminating in a drastically reduced response time alongside enhanced responsivity.

Structurally, the uniquely integrated counterclockwise spiral antenna concurrently serves as the source, drain, and top gate. This monolithic configuration maximizes the spatial symmetry breaking of the localized THz near-field at the nanoscale. Relative to conventional p-n junctions or elementary asymmetric contact geometries, the near-field focusing modulated by the spiral architecture induces an exceptionally steep localized temperature gradient (∇T). Governed by the Seebeck effect, this pronounced spatial asymmetry rigorously dictates the directional diffusion of hot carriers, thereby optimizing the photothermoelectric (PTE) conversion efficiency and yielding a robust macroscopic photocurrent even under zero-bias conditions.

## 4. Conclusions

In summary, this work presents a significant advancement in room-temperature THz photodetection through the successful integration of a spiral-antenna-coupled architecture with large-area monolayer graphene. Validated by both FDTD theoretical simulations and experimental measurements, the macroscopic photoresponse is fundamentally governed by the photothermoelectric (PTE) effect. The spatial symmetry breaking introduced by the spiral structure induces an exceptionally steep localized temperature gradient, which strictly dictates the directional diffusion of hot carriers toward the colder region to generate a robust net photocurrent. A comprehensive evaluation of the temporal dynamics was conducted, wherein the achieved microsecond-scale response time (11 μs) rigorously corroborates the ultrafast nature of this hot-carrier transport mechanism. Ultimately, this meticulous approach to structural and material integration provides critical insights into the fundamental design of high-sensitivity, room-temperature-operating THz photodetectors. Furthermore, the findings of this study highlight the immense potential of antenna-coupled 2D material architectures in fulfilling the rigorous demands of next-generation practical applications, marking a notable step forward for high-speed wireless communications, environmental monitoring, and high-resolution spectroscopy.

## Figures and Tables

**Table 1:** Comparison of THz photodetectors based on different active materials.

Active Materials	Responsivity	Response Time	Ref.
Graphene	3.11 mA/W	11 μs	this work
3D microporous graphene	5.1 mV/W	23 ms	[39]
Black phosphorus	0.35 mA/W	40 μs	[40]
SrTiO_3_	1.18 V/W	1.5 s	[41]
Carbon nanotube film	45 mV/W	80 μs	[42]
Semi-suspended graphene	142 mV/W	100 ms	[43]

## Data Availability

The datasets used and/or analyzed during the current study are available from the corresponding author on reasonable request.

## Supplementary Materials

The following supporting information can be downloaded at: https://www.mdpi.com/article/10.3390/s26092633/s1, Figure S1: Dimensions of the Spiral Antenna; Figure S2: Comparison of left-handed and right-handed helical antennas; Figure S3: Three design schemes of the left-handed spiral structure.

## Abbreviations

The following abbreviations are used in this manuscript:

GFETs	Graphene field-effect transistors
PTE	Photothermoelectric
NEP	Noise equivalent power
THz	Terahertz
ROICs	Readout integrated circuits
CVD	Chemical vapor deposition
FDTD	Finite-difference time-domain
ALD	Atomic layer deposition
PECs	Perfect electric conductors
PMLs	Perfectly matched layers
CNP	Charge neutrality point
