# Supplementary material for: High-Performance Terahertz Photodetectors Based on Spiral Structure-Regulated Graphene
Source: Sensors (Basel). 2026 Apr 24;26(9):2633. doi: 10.3390/s26092633 (PMC13165699; doi:10.3390/s26092633)
Supplement: Supplementary file 1 [file sensors-26-02633-s001.zip › sensors-4234475-supplementary.pdf]

Supplementary Information

High-performance terahertz photodetectors based on spiral structure-regulated graphene

Lei Yang [1], Bohan Zhang [3], Yingdong Wei [2,4], Hongfei Wu [4],Zhiyuan Zhou [5], Zhaowen Bao [4], Huichuan Fan [2], Xiaoyun Wang [2],and Lin Wang [2,*], Xiaoshuang Chen [2,4,*]

[1] School of Microelectronics Shanghai University, 20 Chengzhong Road, Shanghai 201899, China.;yangl23@shu.edu.cn (L.Y.);

[2] State Key Laboratory of Infrared Physics, Shanghai Institute of Technical Physics, Chinese Academy of Sciences, 500 Yu Tian Road, Shanghai 200083, China.; weiyd@shanghaitech.edu.cn (Y.W.); fanhuichuan23@mails.ucas.ac.cn (H.F.);wangxiaoyun23@mails.ucas.ac.cn (X.W.);

[3] School of Information Science and Technology, ShanghaiTech University, Shanghai 201210, China.; zhangbh2023@shanghaitech.edu.cn (B.Z.);

[4] School of Physical Science and Technology, ShanghaiTech University, Shanghai 201210, China; wuhf2023@shanghaitech.edu.cn (H.W.); baozhw2023@shanghaitech.edu.cn (Z.B.);

[5] School of physics, Donghua University, Shanghai, 201620, China.; zhouzhou66321057@163.com (Z.Z.);

* Correspondence: wanglin@mail.sitp.ac.cn (L.W.); xschen@mail.sitp.ac.cn (X.C.);

Supplementary 1:

Inner Spiral Arm: Initiates at a radial distance of 9.5 μm from the center and spirals outward, terminating at 99.5 μm.Outer Spiral Arm: Initiates further out at 17.5 μm and extends to 107.5 μm. Among them, r_1 is 9.5 μm, r_2 is 99.5 μm, r_3 is 17.5 μm, and r_4 is 107.5 μm. As illustrated in the figure below:S1

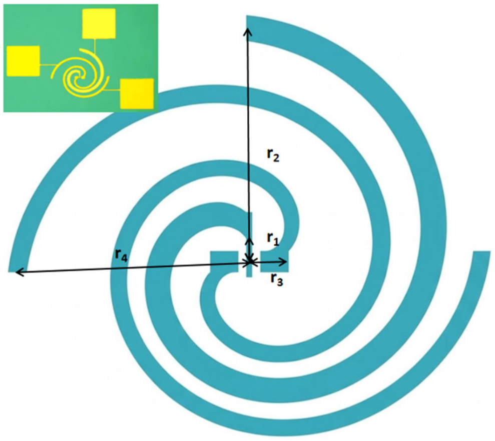

Figure S1: Dimensions of the Spiral Antenna.

Supplementary 2:

Figure S2 shows the designed geometry and the local plasmon-induced electric field intensity distribution in the central region, corresponding to metallic spiral structures with different chiralities (left-handed and right-handed), verifying the efficient optical field enhancement and asymmetric spiral-plasmon coupling capability. The asymmetric electric field distribution leads to variations in the local photon absorption rate, thereby generating a significant temperature gradient for the photoelectric process. Unlike conventional Archimedean spiral antennas, a dual-arm structure is arranged at both ends of the channel, with the third arm acting as a gate finger structure. Ideally, such antennas require a broad spectral operating range and excellent radiation coupling efficiency; however, limited by the size effect of plasmon propagation, this property remains a long-term technical challenge.

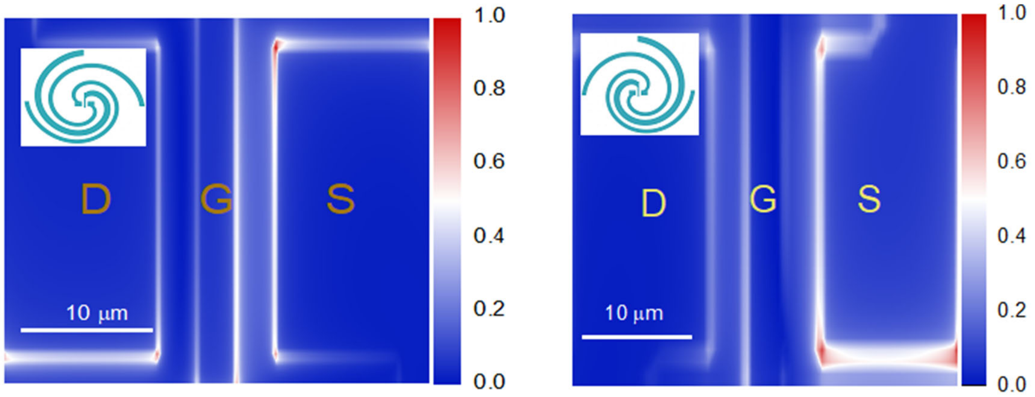

Figure S2: Simulated electric field intensity distributions of the left-handed and right-handed Archimedean spiral antennas. The labels D, G, and S denote the sleeve structures acting as the drain, gate, and source electrodes in the spiral architecture, respectively.

Supplementary 3:

To further verify the effect of the spiral gate finger structure, we designed three left-handed spiral configurations, as shown in the following figures. When the gate finger structure is positioned close to one of the electrodes, the electric field intensity is significantly enhanced.The design with the gate in the middle is adopted in this work. Although simulations indicate that placing the gate near the electrode can further improve the local electric field intensity, the centered design ensures complete and uniform modulation of the Fermi level across the entire channel region by the gate voltage, thereby achieving better modulation depth. It also reduces parasitic capacitance,

which helps maintain the fast response of the detector at 11 μs. This design achieves an optimal balance between detection performance and device reliability while preserving the geometric asymmetry induced by the spiral structure. As shown in Figure S3 below:In the left panel, the gate electrode is 3 μm away from both the source and drain electrodes.In the middle panel, the gate is 2 μm from the drain electrode and 5 μm from the source electrode.Conversely, in the third panel, the gate is 2 μm from the source electrode and 5 μm from the drain electrode.

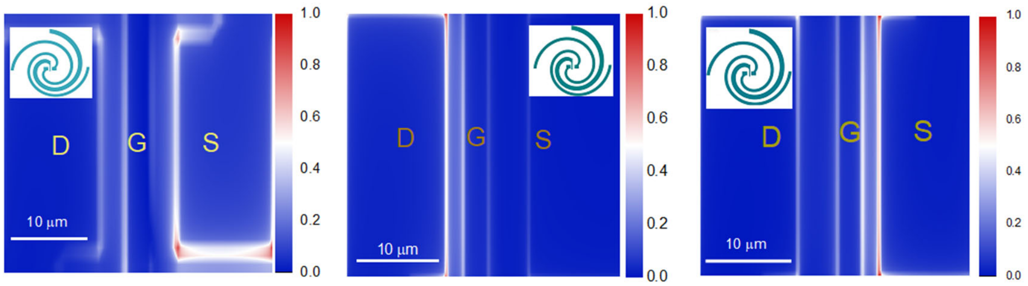

Figure S3: Three design schemes of the left-handed spiral structure.
